# Experimental Pathology—Rational Principles of Therapeutics

**Published:** 1860-08

**Authors:** Claude Bernard

**Affiliations:** Member of the French Institute, and Professor of General Physiology at the Faculty of Sciences


### EXPERIMENTAL PATHOLOGY—RATIONAL PRINCIPLES OF THERAPEUTICS.

*Lecture xii. Delivered at the College of France, during the Winter Session*, 1859-60, *by* M. CLAUDE BERNARD, *Member of the French Institute, and Professor of General Physiology at the Faculty of Sciences.*

GENTLEMEN—We have considered the two opposite points of view from which medical men regard the healing art; one class, as we have seen, recognize the existence of a *vis medicatrix naturæ*, or healing power in nature, to the influence of which they invariably attribute the return of the sick to health; the other class, indignantly rejecting this hypothesis ascribe the honor almost exclusively to the medical man of all the cures which happen to be effected; and these two different opinions, it is not difficult to foresee, must of necessity become apparent in practice. The partisans of the *vis medicatrix naturæ* maintain the principle of expectation, while their adversaries have recourse to a practice more or less energetic and varied.

Both parties have, to a certain extent, truth on their side; though it would be dangerous for medical men to attach themselves exclusively either to the one or to the other opinion. It is true that nature frequently exerts herself in the cure of diseases, but her efforts, often impotent and ill-directed, stand in need of the assistance of art.

It is by means of medicinal agents that man interferes, for the purpose of modifying the course of disease; it is by means of medicinal agents that he accelerates or retards its progress; it is, therefore, indispensible for every medical practitioner who desires to be unfettered by the trammels of a slavish empiricism, to ascertain their precise action, in order that he may be in a suitable position to use them when an occasion shall present itself.

But scarcely have we embarked on the consideration of this subject, when a new question presents itself, and which it becomes our duty to solve before proceeding farther. What are medicines; and what is the difference which separates them from aliments and from poisons? On this subject the following definition is usually accepted: medicines serve to re-establish health; aliments serve to support life; poisons destroy it.

But we are evidently, in this respect, under the necessity of confining ourselves to generalities somewhat vague. Every definition which is of too precise a nature, is apt, on that very account, to become inexact. Not to spend too much time on this point, we will merely say that medicines are foreign substances, introduced into the economy there to determine such and such phenomena; it is, therefore, the nature of their action which it is our duty to specify. We see from this, that all medicines are in their nature poisons, the only difference between the two consisting in the extent of their action; for they act only in virtue of their toxic properties, being naturally substances foreign to the economy. Those substances which already exist in the physiological state in the system, are null in their effects, being neither useful or otherwise in the re-establishment of health when it is deranged.

We know, however, that chlorine, iron, phosphorus, and many other substances, for example, usually employed as medicines, already exist in the body; but they are never found there in a state similar to that in which they are exhibited as medicines. Phosphorus, in its pure state, is an energetic poison; whereas, phosphates are found in abundance in the normal condition of the body. Thus, therefore, all substances, whether medicinal or toxic, are foreign to the system, either by their nature, or in consequence of the particular form in which they are prescribed.

As the greater part of poisons belong either to the mineral or the vegetable kingdom, it is not surprising that medicine borrows the greater number of the agents it employs from the one or the other of these two. Sometimes these agents are vegetable alkaloids, sometimes they are purgative salts, sometimes they are metallic salts; now these different substances, once introduced into the economy, exercise on it a perfectly well-defined action; the salts of quinine combat the periodicity of diseases; mercurial compounds exercise a specific influence over certain virulent affections; how, then, are we to understand the *modus operandi* of each medicinal agent? For a long time it was believed that the medicinal substance, penetrating to the interior of our organs, addressed itself directly to the morbific principle, with a view to neutralizing it; mercury addressing itself to the syphilitic virus, acids to the principle which generates scurvy, and alkalis to that on which rheumatism depends; an attempt has even been made to treat lead colic by the administration of sulphuric acid to those suffering under that affection, with a view to rendering the morbific agent insoluble, and consequently inert in the body of the tissues. These examples will suffice to enable you to understand what has been attempted to be realized by the aid of medicines.

It is easy to prove, by the simplest possible physiological notions, that this theory is altogether false. It is manifestly impossible to produce the sulphate of lead in the torrent of the circulation, and it is equally impossible to render the blood acid, for the animal dies a long time before the circulating fluid has ceased to be alkaline; in fact, in all animals, whether red or white blooded, this fluid is invariably alkaline, although it becomes acid spontaneously after death, when the sugar in it becomes changed into lactic acid.

The chemical combinations which are usually observed to take place in our laboratories, cannot be produced in the blood, but actions of a different kind are readily produced in this fluid; I allude to fermentations. Introduce yeast into the veins, and you will see alcoholic fermentation follow in spite of all the conditions inherent in the vital principle. But fermentations are not, as we know, chemical combinations; they are simply actions. Yeast does not combine with the sugar, it only decomposes it, and herein lies the secret of its poisonous action.

It will be easy to give you a still more striking example of these particular re-actions. We know that amygdaline, a substance of organic origin which is found in the bitter almond, has the property, in presence of certain ferments, of undergoing double decomposition, giving origin to glycose and hydrocyanic acid. Emulsine, the particular ferment which decomposes the amygdaline, exists in the sweet as well as in the bitter almond; but this latter alone contains the principle of amygdaline; hence the characteristic difference, which we know so well, between the odor and flavor of these two fruits, which in every other respect resemble each other.

Let us now suppose that we inject into the veins of an animal either amygdaline or emulsine separately, no accident follows; but if we inject these two substances simultaneously into the blood, at two different points, more or less distant, the one from the other, the animal almost immediately dies, as if struck with lightning; the reaction which brings about the decomposition of the amygdaline is effected in the blood contained in the veins; prussic acid has been engendered, and its poisonous effects have not been slow in manifesting themselves. Here, then, is a reaction to which the presence of albumen, of fibrine, and other substances held in solution in the blood, have offered no obstacle—conclusive evidence that, in opposing themselves to the ordinary chemical reactions, the albuminous substances of the blood act simply in virtue of their chemical properties, and do not, in this respect, exercise any vital influence. If it were possible, by introducing into the blood certain ferments for the purpose of determining reactions of an analogous kind, but of a nature not unfavorable to health, the medical art would doubtless have availed itself of this method of action; but it is unfortunately impossible to do so in practice—in fact, ferments are not generally capable of absorption; at least, up to the present time, we do not know of any one that can be taken up by the stomach; it would be necessary to introduce them directly into the blood. The action of emulsine on amygdaline again furnishes a proof of this. Inject some amygdaline into the veins of an animal, while at the same time you introduce emulsine into the stomach, no reaction whatever takes place, for an impassable barrier opposes the passage of the emulsine into the blood. But when the two substances are simultaneously introduced into the stomach, so that they there combine, reaction follows, prussic acid is set at liberty, and the animal dies of poison. This is what takes place ninety-nine times in a hundred to those who are guilty of the imprudence of eating a large quantity of bitter almonds. If, however, half an hour should elapse between the ingestion of emulsine and that of amygdaline into the stomach of a dog, no accident is observed to take place; the ferment digested in the stomach has descended into the intestines, and has lost all its characteristic properties. We do not, up to the present moment, know any ferment susceptible of being absorbed by the digestive tube.

It is, therefore, impossible to explain the action of medicines on purely chemical grounds; an explanation has, consequently been sought for by appealing to notions borrowed from the physical sciences. It is thus, for example, that an attempt has been made to explain the action of diuretics. M. Poiseuille has proved that if distilled water flows with a given rapidity in capillary tubes, we can, without modifying the conditions of temperature or pressure, increase or diminish the rapidity of the flow by the addition of certain substances. If, for example, we add to distilled water a small proportion of hydroclorate of ammonia, of nitrate of potash, or of some salt of iodine, the flow becomes accelerated; alcohol, the sulphate of soda, and several chlorides, produce the opposite effect of this. M. Poiseuille thought that the diuretic action of the nitrate of potash could be explained by the increased rapidity in the circulation in the capillaries of the kidneys to which it gives rise; while alcoholic intoxication could, he thought, be explained by the diminished rapidity of the cerebral circulation, and might be dissipated by the salts of ammonia, in virtue of the acceleration they produce in the movement of the blood in the capillaries. In repeating his experiments, whether on individual organs of the body or on living animals, M. Poiseuille was able to satisfy himself of the fact that the capillary circulation was influenced by sundry agents which accelerate or retard the flow of water in glass tubes.

These are not the only examples of this nature that might be cited; for instance, an attempt was made to explain the special action of purgatives, which give rise to a very abundant serous discharge from the surface of the intestines by the simple principle of endosmose; it is true the sulphate of soda, and several other saline purgatives, possess endosmotic properties in a very high degree; therefore, it cannot be denied that in many of these cases, the reasoning is somewhat specious; but there are other cases, much more numerous, where this augmentation does not hold good. Purgatives, for instance, in many cases, present but a very feeble endosmotic property, the greater part of the drastic kind being derived from the vegetable kingdom.

There is a third way in which the action of medicines may be understood, and which is more in accordance with physiology. It is admitted that there exists in each organ an elective action, which renders it more apt than the others to be influenced by certain medicinal agents. The different ways by which a substance, introduced into the economy, can escape from it, are, as we have seen, extremely variable. Well, then, medicine acts precisely on that organ by which it is eliminated. Reasoning thus supposes that the medical action is essentially different from the toxic one; such a manner of viewing the question is, however, quite erroneous, as we shall see by and by; but in order to better discuss the theory to which we have just alluded, let us proceed to consider it on scientific grounds. According to this hypothesis, the substances introduced into the economy exercise on the organs which serve for their elimination an action altogether specific, and experimental physiology furnishes us with numerous examples of this.

Ether, we know, when it penetrates, by no matter what passage, into the torrent of the circulation, escapes by the lungs; the characteristic odor of the breath proves this. Well, then, if the quantity of ether intruduced into the economy be sufficient to bring about toxic effects, we find at the autopsy traces of an action altogether local, which has been exercised on the lungs.

But, in the case of phosphorus, the fact is still more evident. It is generally by dissolving the substance in oil that we obtain it in a convenient form for injection into the veins; and when the dose is not of sufficient quantity to produce toxic effects immediately, recourse may be had to the following experiment in order to demonstrate that it is eliminated by the lungs: Place the animal in a dark room, and you will see flames escaping from the mouth and nostrils; this is the well known result of the phosphoresence of the gases given forth by the lungs. When, after having subjected the animal to this experiment, we kill it, we discover in the lungs lesions of the most serious description; everywhere their tissues are congested, and a peculiar yellow hepatization is remarked at different points.

There are, besides, several other examples of a particular action exercises by different medicinal agents on the organs which they traverse on making their escape from the economy. Almost all the secreting organs are capable of being modified in a particular way, by the action of certain substances found in their secretions. We shall content ourselves by pointing out the influence which is almost immediately exercised by cantharides on the urinary organs. We know, in fact, that such is the sensibility of the organs in this respect, that the simple application of the blister suffices, in certain subjects, to bring about a very well-marked irritation in the entire urinary apparatus. The effects of cantharides are still more marked when the substance is taken into the stomach; it then gives rise to inflammation of the bladder, and to acute nephritis, which are not unfrequently followed by the abundant production of false membranes, and in some cases by accidents of the most serious kind.

But in this entire series of phenomena we cannot recognize any other than a purely local action exercised on the particular organs affected. At the time phosphorus and sulphuretted hydrogen traverse the lungs in making their exit from the body, it is found that they have lost nothing of their irritating properties; it is not, therefore, surprisiug that they develope in the center of the organ itself, which serves for their elimination, an inflammation more or less intense. The same holds good as regards cantharides; this substance in traversing the urinay apparatus, acts as a blister on the mucous membrane which lines its internal surface, and there determines acute inflammation.—*Medical Times and Gazette.*

